# Comparative cytotoxicity evaluation of eight root canal sealers

**DOI:** 10.4317/jced.53724

**Published:** 2017-04-01

**Authors:** Claudio Poggio, Paolo Riva, Marco Chiesa, Marco Colombo, Giampiero Pietrocola

**Affiliations:** 1 Department of Clinical-Surgical, Diagnostic and Pediatric Sciences – Section of Dentistry, University of Pavia, Pavia, Italy; 2 Departement of Molecular Medicine, Unit of Biochemistry, University of Pavia, Pavia, Italy

## Abstract

**Background:**

The aim of the present study is to evaluate and compare the cytotoxic effects of eight root canal sealers (BioRoot RCS, TotalFill BC Sealer, MTA Fillapex, Sealapex, AH Plus, EasySeal, Pulp Canal Sealer, N2) on immortalized human gingival fibroblasts over a period of 24, 48 and 72 hours.

**Material and Methods:**

Immortalized human gingival fibroblast-1 HGF-1 (ATCC CRL-2014) were incubated. Root canal sealers were then placed into sterile, cylindrical Teflon moulds. The extraction was made eluting the sealers in cell culture medium. Cells (1 × 104) were seeded in each well of a 96-well plate and incubated for 24 h at 37°C. Cultures were then exposed to 100 μL of the extracts medium. The percentage of viable cells in each well was calculated relative to control cells set to 100%.

**Results:**

BioRoot RCS and TotalFill BC Sealer extracted for 24h showed no cytotoxic effect, while it was mild by using 48 and 72 h extracts. No cytotoxic effect was measured by using AH Plus medium eluted for 24 h, while it was moderate after 48 h and severe after 72 h. Pulp Canal Sealer, Sealapex and N2 showed moderately cytotoxic activity for all the extraction times. EasySeal and MTA Fillapex remained severely or borderline mildly cytotoxic for all the extraction times.

**Conclusions:**

In the present study only BioRoot RCS, TotalFill BC Sealer and AH Plus showed no cytotoxic effects at least in the first 24h. All the other sealers revealed moderately or severely cytotoxic activity during all the extraction times.

** Key words:**Cytotoxicity, gingival fibroblast, MTT test, root canal sealer.

## Introduction

The obturation of root canal systems is one of the most important steps of endodontic treatment. The procedure consists in the three-dimensional filling of the endodontic space in order to prevent the apical and coronal infiltration and the proliferation of microorganisms. Root canals are traditionally filled with gutta-percha points and a root canal sealer. It is widely recognized that sealers if extruded through the apical constriction, may come in direct contact with periapical tissues and may affect them (1,2). Thus, root canal sealers should be non-cytotoxic and biocompatible with periapical tissues (3).

The aim of the present study is to evaluate and compare the cytotoxicity effects of eight root canal sealers on immortalized human gingival fibroblasts over a period of 24, 48 and 72 hours.

## Material and Methods

Eigth root canal sealers were selected for this study: BioRoot RCS/silicate-based sealer (Septodont, Saint-Maur-des-Fosses, France), TotalFill BC Sealer/bioceramic-based sealer (FKG Dentaire SA, La Chaux de Fonds, Switzerland), EasySeal/resin-based sealer (Komet, Lemgo, Germany), MTA Fillapex/MTA-based sealer (Angelus Dental, Londrina, PR, Brazil), Pulp Canal Sea-ler/zinc oxide-eugenol sealer (Kerr, Orange, CA, U.S.A), Sealapex/polymeric calcium hydroxide sealer (Kerr, Orange, CA, U.S.A), N2/zinc oxide-eugenol sealer (Ghimas, Casalecchio di Reno, BO, Italy), AH Plus/resin-based sealer (Dentsply-DeTrey, Konstanz, Germany).

-Cell culture

Immortalized human gingival fibroblast-1 HGF-1 (ATCC CRL-2014) were obtained from the American Type Culture Collection and cultured in high glucose Dulbecco’s modified Eagle’s medium (DMEM; Sigma-Aldrich, St. Louis, MO, USA) supplemented with 4 mM L-glutamine (Sigma-Aldrich), 1% penicillin, streptomycin (Sigma-Aldrich) and 10% (vol/vol) heat-inactivated fetal bovine serum (FBS; Sigma-Aldrich). Cells were incubated at 37°C in 5% CO2 atmosphere, fed every 48 h and routinely sub-cultured every 5 -days with a split ratio of 1:3 using trypsin-EDTA (0.05%; Sigma-Aldrich) for 3 min at 37°C.

-Sample preparation 

Root canal sealers were prepared according to the manufacturer’s recommendation. The sealers were then placed into sterile, cylindrical Teflon moulds which had 4 mm diameter and 2 mm height. Excess material was removed with a sterile scalpel and the sealers were carefully removed from Teflon blocks after setting. To prevent contamination, specimens were exposed to UV light for 24 hours after manipulation. Each sealer was immersed in extraction medium immediately after setting.

-Preparation of the extract

The extraction was made eluting the sealers in cell culture medium (see cell culture paragraph) using the surface area-to-volume ratio of approximately 1.25cm²/ml between the surface of the samples and the volume of medium (4). The extraction vials were the incubated at 37°C for 24 hours, 48 hours or 72 hours. The specimens were then discarded and the elute extracts were filtered by 0.22-μm pore size membranes (Millipore; Billerica, MA, USA). Control samples containing only culture medium were similarly treated. Undiluted extracts were used for the testing.

-Cytotoxicity Test

Cells (1 × 104) were seeded in each well of a 96-well plate and incubated for 24 h at 37°C. Cultures were then exposed to 100 μL of the extracts medium. Cell cultures with supplemented DMEM (FBS and antibiotics solution) were used as controls. After 24 h, cell viability was determined using the MTT assay. The MTT solution (3-{4,5-dimethylthiazol-2-yl}-2,5-diphenyl tetrazolium bromide) (Sigma-Aldrich) in RPMI-1640 without phenol red (Sigma-Aldrich) (5 mg/mL) was added to each well of culture plate to make final concentration of 0.5 mg/mL and the cells were incubated for 4 h at 37°C. Then, the supernatant was removed and the resulting formazan was dissolved by adding 100 μL DMSO (Sigma-Aldrich) to each well. The optical density of formazan dye was read at 545 nm against 620 nm as background by Elisa reader (Bio-Rad, Hercules, California, USA). The percentage of viable cells in each well was calculated relative to control cells set to 100%. Cytotoxicity responses were rated as severe (30%), moderate (30-60%), mild (60-90%) or noncytotoxic (>90%) (5).

## Results

**Figure 1:**
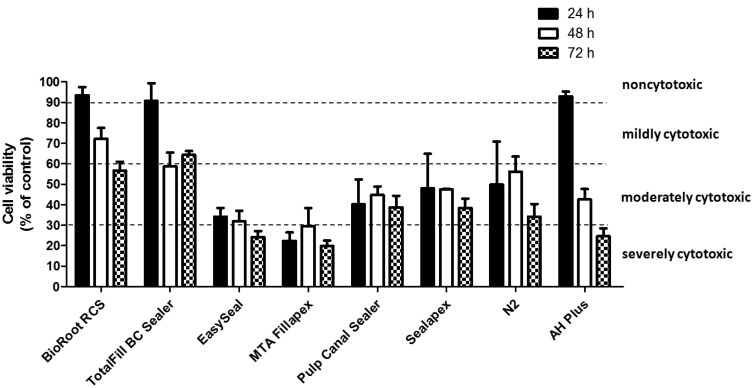
Cell viability in the presence of the elute extracts from eight root canal sealers. Confluent human gingival fibroblast were treated for 24 hours with extracted medium made eluting the sealers for 24 hours, 48 hours or 72 hours. The cell viability was measured by the MTT assay. Values are expressed as percentages relative to the control group and classified as severe (<30%), moderate (<60%), mild (60-90%) or non-cytotoxic (>90%). Bars and error bars represent the means and ± SD from three independent determinations performed in triplicate.

To evaluate cell viability in the presence of the extract from eight root canal sealers, a MTT assay was performed. The results obtained following cell treatment with the extracts are shown in  Table 1 and collectively represented in figure 1.

**Table 1:**
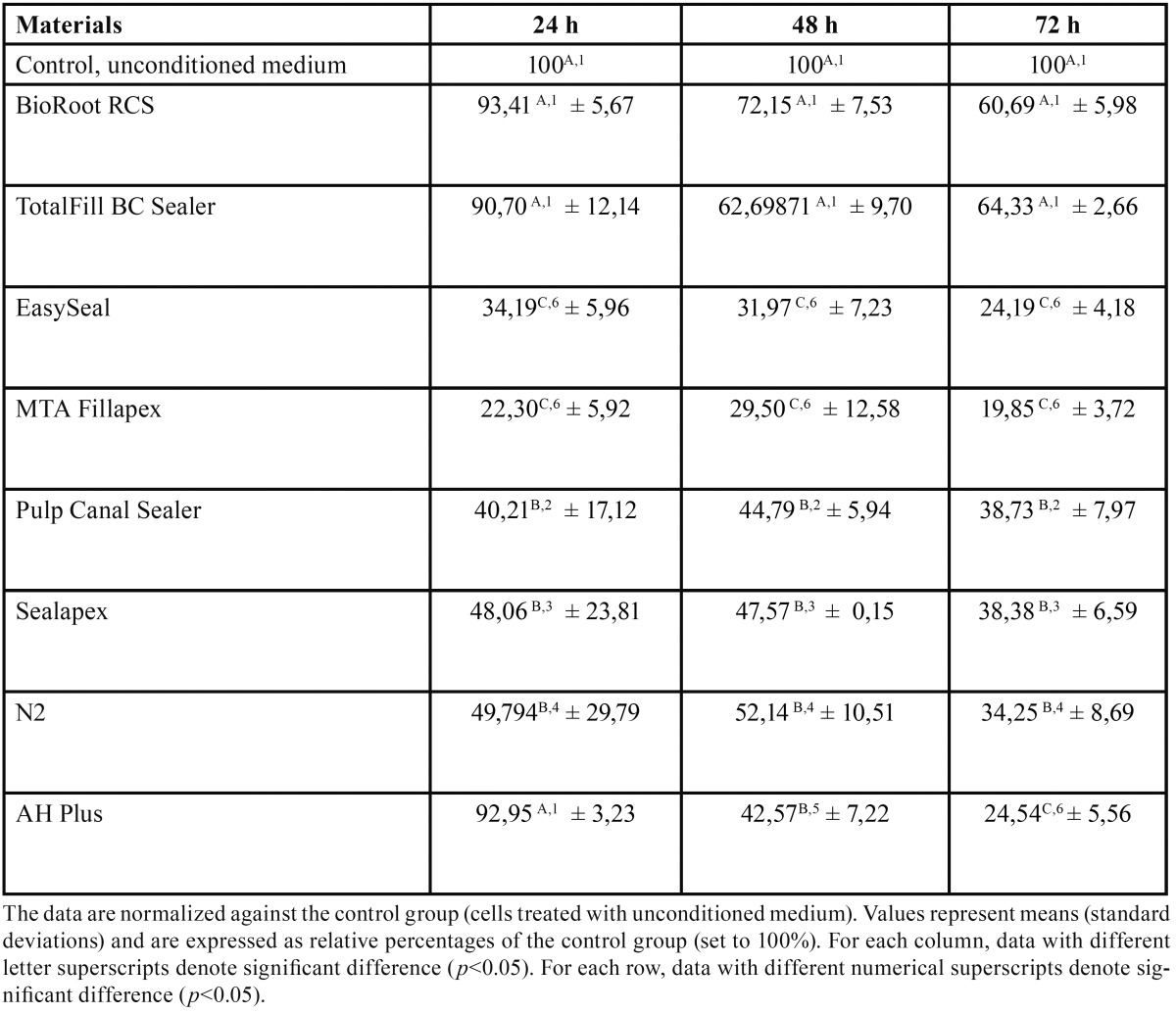
Cell viability in the presence of the elute extracts from eight root canal sealers.

BioRoot RCS and TotalFill BC Sealer extracted for 24h showed no cytotoxic effect, while it was mild by using 48 and 72 h extracts. However, differences in cytotoxicity for all the times were not statistically significant (*p*>0.05) compared to the control (culture medium only).

No cytotoxic effect was measured by using AH Plus medium eluted for 24 h, while it was moderate after 48 h and severe after 72 h. The differences in cell viability in the last two extraction times were statistically significant compare to the control (*p*<0.05). Moreover, differences in viability of the cells treated with 48 h or 72 h extraction medium were also statistically significant (*p*<0.05).

Pulp Canal Sealer, Sealapex and N2 showed moderately cytotoxic activity for all the extraction times. Their differences in cytotoxicity were also statistically significant compare to the control (*p*<0.05).

EasySeal and MTA Fillapex remained severely or borderline mildly cytotoxic for all the extraction times. After 72 h of elution, both sealers exhibited a toxicity level that was significantly more severe (*p*<0.05) than the other tested sealers.

When cytotoxicity was moderate or severe, cells died by apoptosis (data not shown). Rounding and detachment of the cells from the plastic wells are the typical apoptotic events observed by optical light microscope.

## Discussion

Root canal sealers should be biocompatible because they might extrude thought the apical constriction and contact intimately the soft periodontal tissue. When out of the root canal, a sealer could induce cytotoxic damage to tissue and have different level of cytotoxicity over a period of 24, 48 or 72 hours (1,2). Is important to evaluate sealers over different periods after setting because they probably change their cytotoxicity due to diffusion of toxic components resulting from the degradation of the components of the sealers. Freshly mixed materials are analysed because previous reports have shown that the cytotoxicity of sealers is higher immediately after mixing (5,6). Further more it is important to consider the cell type that could be choose for the in vitro biocompatibility study. In this study immortalized human gingival fibroblast are chosen for the close relation between them and endodontic sealers and cements (7,8). The human gingival fibroblasts can be cultured in a low number of passages, resulting in minimal cell changes due to cell culture manipulation (9).

This study is created to determine the cytotoxic properties of eight endodontic sealers on fibroblasts: to evaluate cell viability in the presence of the extract from eight root canal sealers, a MTT assay was performed.

In agreement with our results, BioRoot RCS extracted for 24h shows no cytotoxic effect, while it is mild by using 48 and 72h extracts (10). However, differences in cytotoxicity for all the times are not statistically significant (*p*>0.05) compared to the control (11). Similar results are obtained with TotalFill BC Sealer that has no cytotoxic effect at 24h after setting (12), while it is mild at 48 and 72h. Despite this, as previously said for BioRoot RCS, differences in cytotoxicity for all the times are not statistically significant compared to the control.

No cytotoxic effect is measured by using AH Plus medium eluted for 24 h, while it is moderate after 48 h and severe after 72 h. The current results are partially in accordance with previous studies, which demonstrated the cytotoxic effects of AH Plus (13,14).

EasySeal and MTA Fillapex remain severely or borderline mildly cytotoxic for all the extraction times. After 72 h of elution, both sealers exhibit a toxicity level that is significantly more severe (*p*<0.05) than the other tested sealers. MTA Fillapex was developed in an attempt to combine the physicochemical properties of an endodontic sealer with the excellent biological properties of MTA. According to the present results, MTA Fillapex shows a severe cytotoxicity when cells are exposed to the fresh elutes of the sealer (13,15,16).

MTA Fillapex remains severely and mildly cytotoxic over the entire experimental period. These results suggest correlations between the components, such as salicylate resin and diluting resin with the cytotoxic effects (2).

Similar results are obtained with EasySeal, a resin-based sealer that shows borderline cytotoxicity during all three different extraction times.

Pulp Canal Sealer, Sealapex and N2 show moderately cytotoxic activity for all the extraction times. In agreement with our results, zinc oxide-eugenol-based sealers have been shown to be cytotoxic, which has been attributed to the eugenol present in different formulations.

Biocompatibility of an endodontic sealer is one of the basic conditions for a successful endodontic treatment and healing of the periodontium. So, considerations for choosing an adequate root canal sealer include its physical properties and biocompatibility, but, despite the irritability that endodontic sealers may cause to periapical tissues, endodontists should evaluate the advantages and disadvantages of sealer extrusion (2).

In the present study only BioRoot RCS, TotalFill BC Sealer and AH Plus showed no cytotoxic effects at least in the first 24h. The other sealers tested revealed moderately or severely cytotoxic activity during all the extraction times.
